# Novel Therapeutic Strategies for Active Crohn’s Disease: Targeting Pathobionts and Host–Microbe Interactions

**DOI:** 10.3390/antibiotics15100964

**Published:** 2026-09-30

**Authors:** Gaetano Iaquinto, Errico Picariello, Salvatore Iaquinto, Raffaele Melina, Carmine Sellitto, Simone Sellitto, Giovanna Cataldo, Raffaele Pastore, Vera Rotondi Aufiero

**Affiliations:** 1 Gastroenterology Department, St. Rita Hospital, NefroCenter Group, 83042 Atripalda, Italy; iaquintog@yahoo.it (G.I.); sellitto.simone@spes.uniud.it (S.S.); 2 Institute of Food Sciences, Consiglio Nazionale Delle Ricerche (CNR), 83100 Avellino, Italy; errico.picariello@isa.cnr.it; 3 Gastroenterology Unit, St. Filippo Neri Hospital, 00135 Rome, Italy; salvatore.iaquinto@aslroma1.it; 4 Gastroenterology Unit, San G. Moscati Hospital, 83100 Avellino, Italy; raffaelemelina@icloud.com; 5 Department of Medical and Surgical Specialities and Dentistry, University of Campania Luigi Vanvitelli, 81100 Naples, Italy; carmine.sellito@unicampania.it; 6 Section of Human Anatomy, Department of Mental and Physical Health and Preventive Medicine, University of Campania “Luigi Vanvitelli”, 80138 Naples, Italy; giovanna.cataldo00@outlook.it; 7 Department of Medicine and Health Sciences ‘Vincenzo Tiberio’, University of Molise, 86100 Campobasso, Italy; raffaele.pastore@unimol.it; 8 European Laboratory for the Investigation of Food Induced Diseases, Hosted Within the Pediatric Section of the Department of Medical Translational Sciences Via S. Pansini 5, 80131 Naples, Italy

**Keywords:** Crohn’s disease, adherent-invasive *Escherichia coli*, microbiota, antibiotics, antimicrobial peptides, gut bacteriophages, fecal microbiota transplantation, nutraceuticals, precision medicine, active Crohn’s disease

## Abstract

Crohn’s disease (CD) is a chronic inflammatory disorder characterized by complex interactions among genetic susceptibility, environmental factors, dysregulated immune responses, and alterations of the intestinal microbiota. Although corticosteroids, immunomodulators, biologics, and advanced small-molecule therapies remain central to disease management, a substantial proportion of patients experience primary non-response, loss of response, or treatment-related adverse effects. This has stimulated interest in complementary therapeutic strategies targeting host–microbe interactions and intestinal microbial homeostasis. This review critically examines emerging microbiome-directed approaches for CD, with particular emphasis on pathobionts such as adherent-invasive *Escherichia coli* (AIEC). We discuss the available evidence for antibiotics, antimicrobial peptides (AMP), gut bacteriophages, fecal microbiota transplantation (FMT), and selected nutraceutical and dietary interventions. While some approaches show promising biological or clinical signals, the strength of evidence varies considerably, ranging from in vitro and animal studies to randomized clinical trials and meta-analyses. Antibiotic therapies targeting intestinal pathogens have shown heterogeneous results, suggesting the need for pathogen-guided and intracellularly active antimicrobial strategies. Novel approaches, including AIEC-specific phages and AMP-based interventions, represent promising microbiome-sparing alternatives capable of selectively modulating disease-associated microorganisms. Furthermore, FMT and nutraceuticals may contribute to restoring microbial balance and strengthening intestinal homeostasis, although their efficacy requires further validation in controlled clinical trials. Overall, these strategies support a transition toward precision medicine approaches integrating microbial profiling, host immune characterization, and targeted antimicrobial interventions to improve therapeutic outcomes in CD.

## 1. Introduction

Over the past decades, several microorganisms have been investigated as potential contributors to Crohn’s disease (CD) pathogenesis. Among these, enterohepatic *Helicobacter* species have been associated with Inflammatory Bowel Disease (IBD), with a higher prevalence reported in patients with CD compared with controls [1]. In addition, several pathobionts, including *Yersinia enterocolitica*, have been detected more frequently in the intestinal mucosa of patients with CD, although whether their presence represents a cause or a consequence of the disease-associated intestinal environment remains uncertain [2]. Several studies and meta-analyses using molecular and other microbiological approaches have reported a higher frequency of *Mycobacterium avium* subspecies *paratuberculosis* (MAP) detection in patients with CD compared with controls [3,4,5,6]. However, the pathogenic role of MAP in CD remains controversial, and the available evidence does not establish a causal relationship between MAP infection and disease development.

More recently, attention has been increasingly focused on specific pathobionts within the intestinal microbiota, particularly adherent-invasive *Escherichia coli* (AIEC). AIEC strains are frequently isolated from the intestinal mucosa of patients with CD and possess several pathogenic properties, including adhesion to intestinal epithelial cells, invasion of the epithelial barrier, and survival and replication within macrophages [7,8,9,10]. These characteristics have led to the hypothesis that AIEC may contribute to persistent intestinal inflammation in susceptible individuals. Nevertheless, the presence of AIEC does not by itself establish a causal role in CD, and further studies are required to define its contribution to disease development and progression.

Current treatment strategies for CD primarily aim to control intestinal inflammation through corticosteroids, immunomodulators, biologic agents, and advanced small-molecule therapies. However, a proportion of patients do not achieve an adequate response, experience loss of response over time, or develop treatment-related adverse effects, underscoring the need for additional therapeutic approaches targeting mechanisms beyond conventional immunomodulation [11,12].

This review focuses on emerging microbiome-directed strategies in CD, particularly approaches targeting pathobionts such as AIEC and those aimed at restoring intestinal microbial homeostasis. We critically examined the available evidence for antibiotics, antimicrobial peptides (AMPs), gut bacteriophages, fecal microbiota transplantation (FMT), and selected nutraceutical interventions. Importantly, these approaches differ substantially in their level of development and supporting evidence. Therefore, evidence from in vitro and animal studies is distinguished from findings derived from clinical trials, systematic reviews, and clinical guidelines [12,13].

Rather than considering these interventions as established alternatives to biologic or small-molecule therapies, this review evaluates their potential as emerging or complementary strategies in CD. Emphasis is placed on the limitations of current evidence, the need for appropriate patient selection, and the development of future precision approaches integrating microbial, immunological, and clinical characteristics.

AIEC strains may contribute to the persistence of intestinal inflammation by promoting bacterial colonization and prolonged activation of host immune responses [14,15]. However, although AIEC colonization is consistently associated with CD, the extent to which these bacteria act as primary drivers of disease rather than benefiting from the inflammatory intestinal environment remains to be fully established.

Experimental studies have shown that AIEC infection can promote the production of pro-inflammatory mediators and activate pathways associated with both innate and adaptive immune responses. Increased production of cytokines involved in Th1- and Th17-related responses, including interferon-γ (IFN-γ), tumor necrosis factor-α (TNF-α), interleukin (IL)-12, IL-17A, IL-21, and IL-23, have been reported in experimental settings [16,17]. These findings support the biological plausibility of the role for AIEC in sustaining intestinal inflammation, although experimental observations should not be interpreted as direct evidence of a causal role in human disease.

Bacterial motility and the ability to interact with the intestinal mucus layer may facilitate contact between AIEC and the epithelial surface [18]. Adhesion of AIEC to ileal epithelial cells is mediated, at least in part, by type 1 pili expressing the FimH adhesin and by increased expression of carcinoembryonic antigen-related cell adhesion molecule 6 (CEACAM6) on the apical surface of ileal epithelial cells [19]. CEACAM6 expression has been reported to be increased in the ileal mucosa of patients with CD and may therefore provide a favorable environment for AIEC colonization.

**Figure 1:**
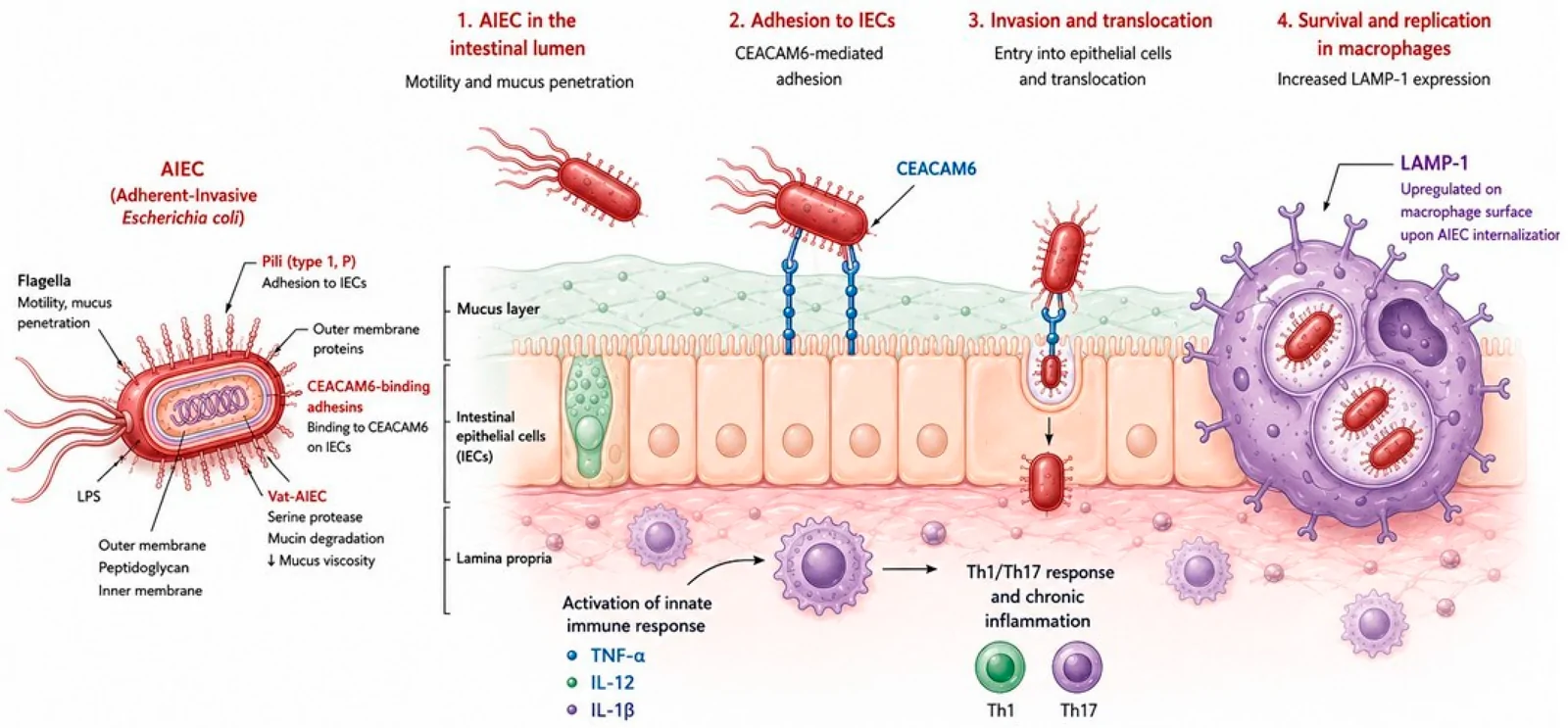
Proposed mechanisms of AIEC pathogenesis in CD. AIEC crosses the mucus layer through flagella-mediated motility and mucus-degrading factors, including Vat-AIEC, and adheres to intestinal epithelial cells via type 1 pili–CEACAM6 interactions. AIEC subsequently invades epithelial cells and translocates into the lamina propria, where it is taken up by macrophages and persists within altered phagolysosome compartments. This promotes LAMP-1 expression and the release of pro-inflammatory cytokines, including TNF-α, IL-1β, and IL-12, contributing to Th1/Th17 responses and chronic intestinal inflammation.

In addition, some AIEC strains express virulence-associated factors that may facilitate mucosal colonization. For example, Vat-AIEC, a serine protease identified in specific AIEC strains, has been reported to alter mucus properties and promote bacterial penetration through the mucus layer [20] (Figure 1).

Importantly, AIEC represents a phenotypically defined pathotype rather than a genetically homogeneous bacterial group, and no single virulence factor is universally present among all AIEC strains. Therefore, the pathogenic potential of AIEC may vary among isolates and according to the host and intestinal environment.

Since their initial description by Darfeuille-Michaud and colleagues in 1998 [7], AIEC strains have been repeatedly isolated from intestinal samples obtained from patients with CD. Several studies have reported an increased prevalence of AIEC in the intestinal mucosa of patients with CD compared with non-IBD controls, particularly in the ileum, although increased adherence and invasion of *Escherichia coli* have also been reported in colonic mucosa [7,9,10,21,22]. Nevertheless, reported prevalence rates vary substantially across studies because of differences in patient populations, sampling sites, bacterial isolation procedures, and the phenotypic methods used to define AIEC.

More recently, the PACIFIC study provided evidence that AIEC-associated phenotypic characteristics may be observed across geographically distinct populations. AIEC was detected in ileal samples from 24.5% of patients with CD in France and 30.0% of those in Hong Kong, with no significant differences in bacterial adhesion or invasion between the two populations [23]. The study also identified differences in antibiotic resistance profiles, while genomic analyses did not identify a single virulence factor specifically associated with AIEC status or geographic origin. These findings support the presence of phenotypically similar AIEC populations across different geographic settings while also highlighting the heterogeneity of this pathotype.

## 2. Current Therapeutic Treatments for Active CD

Current therapeutic strategies for CD include corticosteroids, immunomodulators, biologic agents, and advanced small-molecule therapies. These treatments primarily target dysregulated immune pathways and are central to the induction and maintenance of remission. Nevertheless, a proportion of patients fail to respond adequately to induction therapy or subsequently lose response, highlighting the need for complementary therapeutic approaches targeting additional mechanisms involved in disease pathogenesis.

Growing evidence implicating alterations in the intestinal microbiota and specific pathobionts, including AIEC, has stimulated interest in therapeutic strategies aimed at modulating host–microbe interactions. These approaches include antibiotics, AMPs, gut bacteriophages, FMT, and selected nutraceutical interventions. However, the level of evidence supporting these strategies is highly heterogeneous, ranging from experimental and pre-clinical studies to randomized clinical trials and meta-analyses. Therefore, their potential role in CD should be considered according to the specific clinical setting and the strength of the available evidence.

In the following sections, we critically discuss these emerging approaches, distinguishing established clinical indications from investigational strategies and highlighting the main limitations of the available evidence.

### 2.1. Antibiotics

The potential role of antibiotics in CD is supported in selected clinical settings, including postoperative prophylaxis and the management of specific complications such as perianal disease and intra-abdominal or perianal abscesses (Table 1).

In contrast, the evidence supporting antibiotics for the treatment of active luminal CD is substantially weaker. In particular, nitroimidazole antibiotics such as metronidazole have not consistently demonstrated superiority over placebo for the induction of remission or improvement of mucosal healing in active CD (Table 2) [30,31,32,33,34,35].

**Figure 2:**
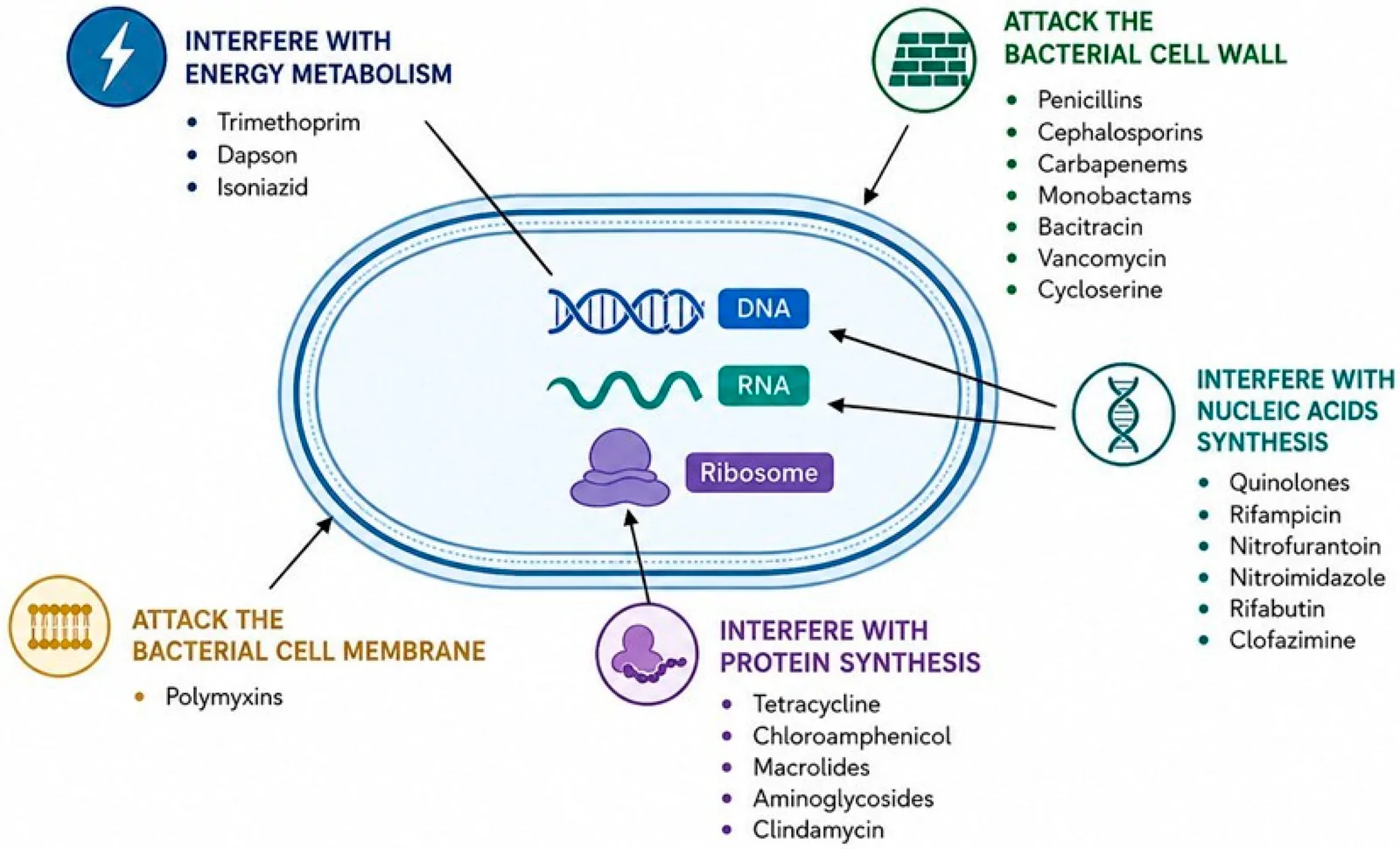
Mechanisms of action of antibiotics against bacterial pathogens. Antibiotics exert their antimicrobial activity by targeting essential bacterial structures and physiological processes required for growth and survival. The principal mechanisms of action include: inhibition of metabolic pathways involved in energy production and biosynthesis; disruption of bacterial cell wall synthesis, leading to loss of structural integrity and cell lysis; interference with nucleic acid synthesis and function through inhibition of DNA replication or RNA transcription; inhibition of protein synthesis by targeting bacterial ribosomal subunits; and disruption of cell membrane integrity, resulting in altered permeability and cell death. Different antibiotic classes act through one or more of these mechanisms, ultimately preventing bacterial proliferation or inducing bacterial killing.

Nevertheless, the involvement of intestinal microorganisms in CD pathogenesis has provided a rationale for investigating antibiotics as therapeutic agents in selected patients with active disease (Figure 2).

The clinical efficacy of antibiotic therapy in active CD is heterogeneous and appears to depend on the disease phenotype, antibiotic regimen, treatment duration, microbiological characteristics, and clinical endpoint assessed. More recently, pathogen-directed strategies have been investigated to specifically target AIEC, a pathobiont associated with ileal CD.

**Figure 3:**
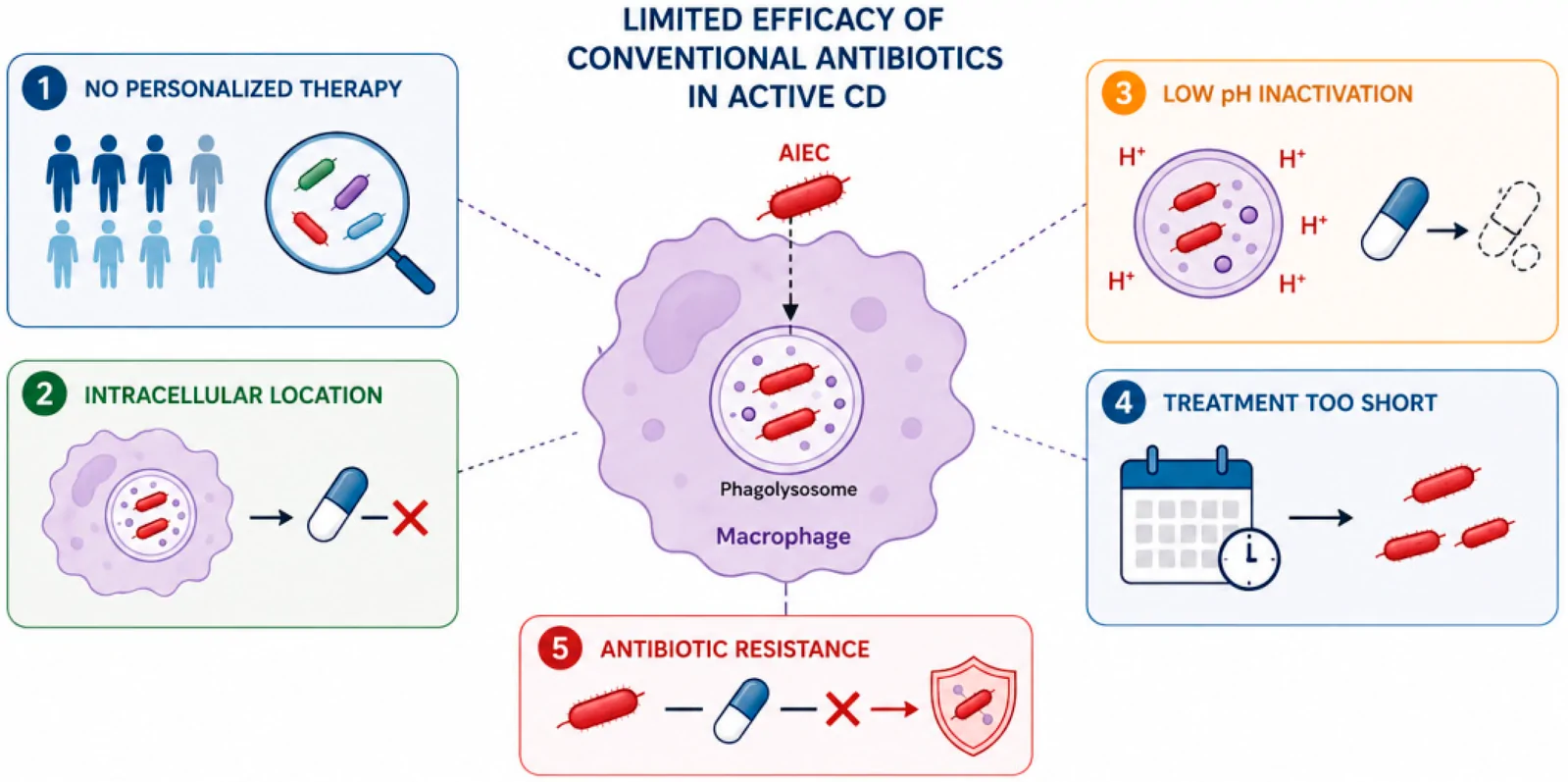
Antibiotic Failure in CD.

The TEOREM trial, published in 2025, evaluated ciprofloxacin plus rifaximin versus placebo for 12 weeks in patients with endoscopically active ileal CD and AIEC colonization. The primary endpoint was endoscopic overall response. Endoscopic response was observed in 50% of patients receiving ciprofloxacin plus rifaximin compared with 33% of those receiving placebo; however, the difference between groups was not statistically significant. Although AIEC clearance was observed in a proportion of patients receiving antibiotic therapy, no significant association was found between AIEC clearance and endoscopic outcomes [36]. Several factors may contribute to the limited efficacy of conventional antibiotic approaches in active CD. First, only a limited number of studies have evaluated microbiologically guided or personalized antibiotic therapy based on the presence of specific bacterial pathobionts. Second, intracellular pathogens such as AIEC can survive and replicate within macrophage phagolysosomes, potentially limiting the efficacy of antibiotics with inadequate intracellular activity [37]. Third, the intracellular environment, including the acidic conditions of phagolysosomes, may influence antibiotic activity and bacterial susceptibility [38]. Additional factors include treatment duration [30,31,32,33,34,35], antimicrobial resistance, and heterogeneity in the detection and definition of AIEC (Figure 3).

Experimental studies have investigated strategies aimed at modifying the intracellular environment to enhance bacterial killing. Flanagan et al. demonstrated that hydroxychloroquine can alkalinize phagolysosomes and enhance intracellular killing of AIEC, potentially increasing the activity of selected antibiotics [39,40]. In a clinical study, long-term combination therapy with hydroxychloroquine, ciprofloxacin, and doxycycline was associated with clinical remission in a subset of patients with active CD [41]. However, these findings remain preliminary and require confirmation in adequately powered randomized clinical trials.

Future antibiotic strategies should therefore focus on better-defined patient populations, microbiological characterization of target pathogens, optimized treatment duration, adequate intracellular drug activity, and pathogen-directed or combination approaches in appropriately selected AIEC-positive patients.

### 2.2. Antimicrobial Peptides (AMPs)

AMPs are important components of the innate immune system and contribute to host defense and intestinal microbial homeostasis. They are generally short peptides, typically ranging from approximately 10 to 100 amino acids, whose antimicrobial activity can be mediated through several mechanisms, including disruption of microbial membranes, interference with intracellular targets, and modulation of microbial metabolic processes. Unlike conventional antibiotics, many AMPs display activity against a broad range of microorganisms; however, their efficacy depends on peptide structure, concentration, ionic conditions, and the characteristics of the target microorganism [42,43].

In the gastrointestinal tract, AMPs are produced by intestinal epithelial cells, particularly Paneth cells, as well as by neutrophils and other immune cell populations. Their functions extend beyond direct antimicrobial activity and include regulation of microbial communities, maintenance of epithelial barrier integrity, and modulation of immune and inflammatory responses [44,45]. Paneth cells are a major source of AMP in the small intestine, particularly α-defensins, which contribute to the regulation of the local microbiota and maintenance of intestinal homeostasis [45,46]. Alterations in AMP expression and Paneth cell function have been described in CD and other forms of inflammatory bowel disease (IBD). Ileal CD is associated with reduced expression of Paneth cell-derived α-defensins and impaired mucosal antimicrobial activity, suggesting that defects in innate antimicrobial defense may contribute to dysbiosis and intestinal inflammation [46,47,48]. However, alterations in AMP expression may also occur because of inflammation, and the relationship between AMP dysregulation and disease pathogenesis remains complex [44,47].

Experimental studies have demonstrated that several endogenous and synthetic AMPs can inhibit the growth of *Escherichia coli* and other clinically relevant bacterial species. Alves et al. [49], for example, demonstrated that the AMP BP100 and pepR induce perturbation and disruption of the *E. coli* cell surface. More recently, Santos-Júnior et al. [50] used a machine-learning-based approach to identify AMP within the global microbiome. From a large computationally predicted repertoire, 100 candidate peptides were synthesized and experimentally tested against clinically relevant drug-resistant pathogens and human gut commensals. A total of 69 peptides displayed antimicrobial activity, including 63 with activity against pathogenic bacteria, supporting the potential of microbiome-derived AMPs as a source of novel antimicrobial agents [50]. Nevertheless, these findings remain predominantly preclinical and cannot be directly extrapolated to therapeutic efficacy in patients with CD.

The principal AMP families involved in intestinal host defense include defensins and cathelicidins. In addition, several antimicrobial proteins contribute to mucosal defense and nutritional immunity, including lactoferrin, calprotectin, and lipocalin-2 [44,51]. Human α- and β-defensins are cationic peptides that exert antimicrobial activity primarily through interactions with microbial membranes, although additional mechanisms have also been described [42,43,52]. Paneth cell-derived α-defensins, including human α-defensin 5 (HD-5) and HD-6, play an important role in controlling the composition of the small-intestinal microbiota and maintaining epithelial homeostasis [45,46].

**Figure 4:**
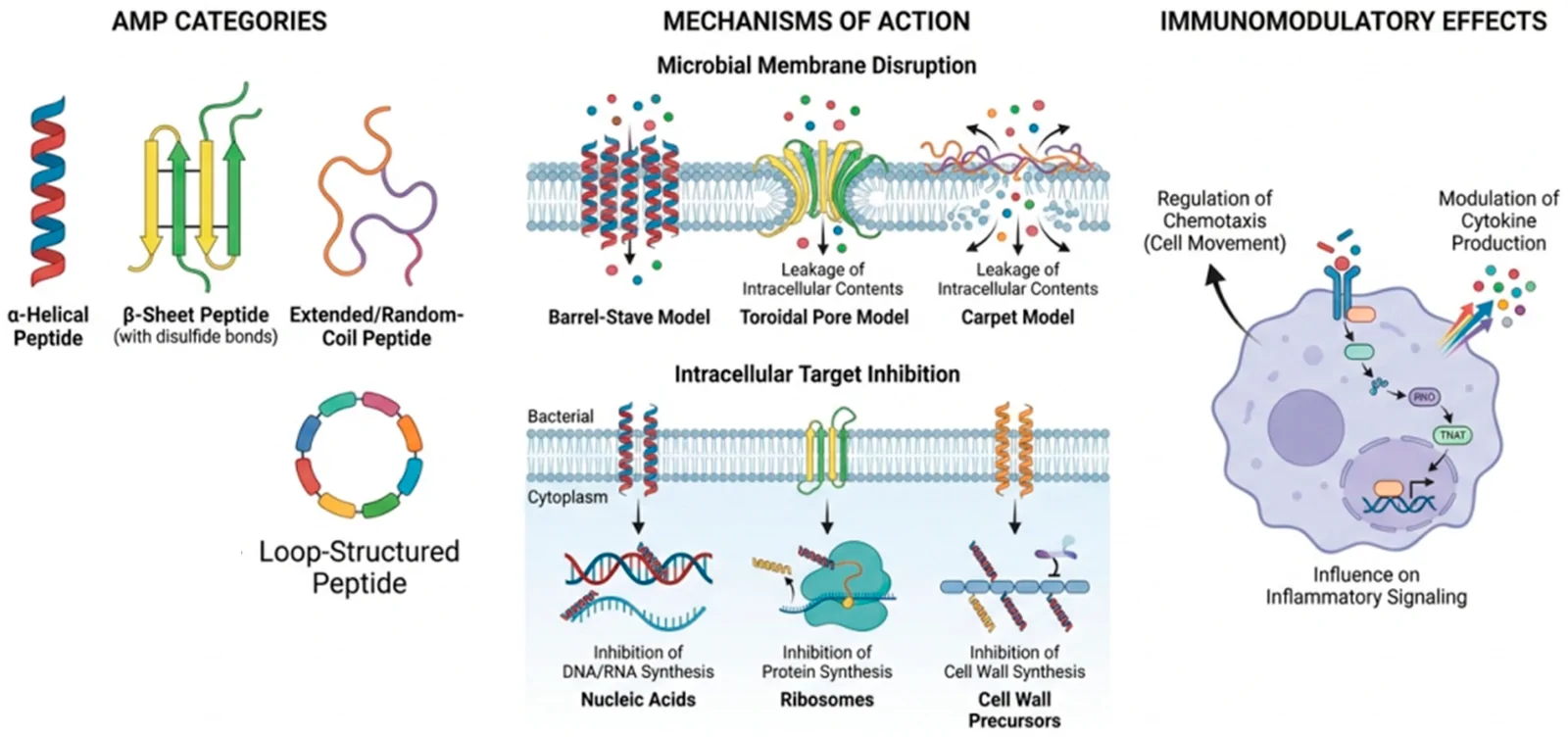
Major classes of AMPs and mechanism of action. AMPs are broadly categorized into α-helical peptides, β-sheet peptides stabilized by disulfide bonds, extended/random-coil peptides, and loop-structured peptides. These molecules exert antimicrobial activity through multiple mechanisms, including disruption of microbial membrane integrity via pore formation (e.g., barrel-stave, toroidal pore, and carpet models), membrane permeabilization leading to leakage of intracellular contents, and inhibition of intracellular targets such as nucleic acid, protein, and cell wall synthesis. In addition to direct microbicidal effects, several AMPs modulate host immune responses by regulating chemotaxis, cytokine production, and inflammatory signalling pathways.

The altered antimicrobial defense observed in CD has provided a rationale for investigating AMPs as potential therapeutic agents. Experimental studies have reported antibacterial and anti-inflammatory effects of selected AMPs in models of intestinal inflammation. For example, the human cathelicidin-derived peptide KR-12 showed antibacterial and anti-inflammatory effects in experimental models of colitis [53]. However, evidence for the therapeutic use of AMPs in CD remains largely preclinical, and their potential clinical application requires further investigation regarding efficacy, stability, toxicity, delivery, and possible effects on the physiological intestinal microbiota (Figure 4).

β-defensins have demonstrated activity against several bacterial species, including *E. coli*, *P. aeruginosa*, *Staphylococcus aureus*, and *Streptococcus pyogenes*. However, antimicrobial activity observed in vitro does not necessarily predict therapeutic efficacy within the complex intestinal environment [52,54,55]. Evidence from experimental models suggests that AMP administration or modulation may influence intestinal inflammation. In chemically induced models of colitis, administration of selected α- and β-defensins has been associated with reduced disease activity, mucosal injury, and pro-inflammatory cytokine expression [56,57,58,59,60]. However, these effects appear to be dose- and context-dependent. For example, Maeda et al. [57] reported that mild transgenic overexpression of human neutrophil peptide-1 (HNP-1) reduced susceptibility to dextran sulfate sodium (DSS)-induced colitis, whereas Hashimoto et al. [58] observed that high-dose administration of defensins exacerbated disease severity in experimental models. These findings highlight the complexity of AMP-mediated immune regulation and indicate that increased AMP activity is not necessarily uniformly beneficial.

Cathelicidin is expressed by several cell types, including epithelial cells and innate immune cells, and contributes to antimicrobial host defense and immunomodulation. Experimental studies have suggested that altered cathelicidin expression may influence susceptibility to bacterial infection and intestinal inflammation. Similarly, exogenous administration of cathelicidin or elafin has shown anti-inflammatory effects in experimental models of intestinal inflammation [61,62]. Nevertheless, these observations remain predominantly preclinical, and evidence supporting the therapeutic administration of these peptides in patients with CD is currently insufficient.

Other molecules involved in nutritional immunity, including lactoferrin, calprotectin, lipocalin-2, and hepcidin, can limit microbial growth by restricting the availability of essential micronutrients. Several of these molecules are also associated with intestinal inflammation and have been investigated as biomarkers of disease activity. For example, circulating hepcidin concentrations have been reported to differ between patients with active IBD and healthy controls [63]. Importantly, their value as biomarkers should be distinguished from their potential therapeutic use, as increased endogenous expression during inflammation does not establish that exogenous administration would be beneficial.

Several AMPs and AMP-related molecules, including calprotectin, defensins, cathelicidin, lactoferrin, hepcidin, and elafin, have been investigated as potential non-invasive biomarkers of intestinal inflammation [64,65,66]. Among these, fecal calprotectin is already widely used in clinical practice for disease monitoring, whereas the clinical utility of several other AMP-related markers remains less well established. Their potential value as therapeutic agents is even more preliminary.

Overall, AMPs represent biologically plausible candidates for microbiome-directed therapeutic strategies because of their antimicrobial and immunomodulatory properties. However, the current evidence supporting their use as direct therapeutic agents in CD is largely derived from in vitro studies and experimental models. Important challenges include peptide stability in the gastrointestinal tract, delivery to the appropriate intestinal site, dose optimization, potential cytotoxicity, effects on commensal microbiota, manufacturing costs, and the risk of unintended immune effects. Therefore, although AMP-based strategies are promising, they should currently be considered investigational, and dedicated clinical studies are required before their therapeutic role in CD can be established.

### 2.3. Gut Bacteriophages

**Figure 5:**
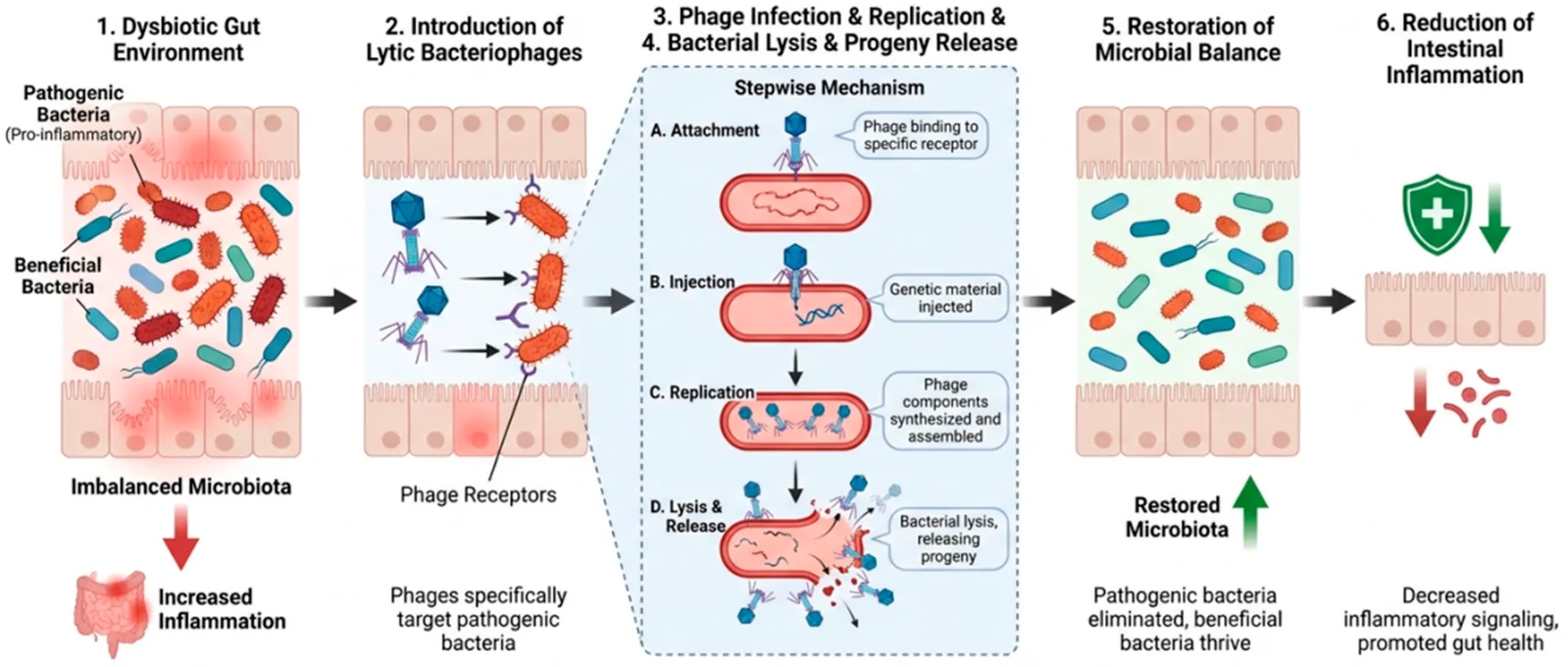
Mechanisms of action of bacteriophages. Lytic bacteriophages selectively target and eliminate pathogenic or dysbiotic bacterial populations implicated in intestinal inflammation. Phages bind to specific bacterial receptors, inject their genetic material, and replicate within the host bacterium, leading to bacterial lysis and release of progeny phages. This process can help restore microbial balance in the gut microbiota and reduce pro-inflammatory bacterial triggers.

Bacteriophages, commonly referred to as phages, are viruses that infect bacteria and represent a major component of microbial ecosystems, including the human gastrointestinal tract [67]. Their potential therapeutic application is based on their ability to selectively infect specific bacterial hosts and, in the case of lytic phages, induce bacterial destruction. Phages recognize specific receptors on the bacterial surface, deliver their genetic material into susceptible bacterial cells, and use host cellular machinery to generate progeny virions, ultimately resulting in bacterial lysis [68] (Figure 5).

The high host specificity of bacteriophages represents a potential advantage over broad-spectrum antibiotics, as phage-based interventions may selectively target pathogenic or disease-associated bacteria while potentially limiting disruption of the commensal microbiota. This property has generated increasing interest in phage therapy as a precision antimicrobial strategy for diseases associated with specific bacterial pathobionts. However, therapeutic efficacy depends on several factors, including phage–host compatibility, bacterial heterogeneity, the emergence of phage resistance, phage delivery and stability, and the complex interactions between phages, bacteria, and the host immune system [68,69,70,71].

Interest in phage therapy for CD has largely focused on the possibility of selectively targeting AIEC. It is important, however, to distinguish evidence obtained using conventional *E. coli* strains or other intestinal pathogens from studies specifically targeting AIEC or evaluating experimental models relevant to CD. Experimental studies using *E. coli*-targeting phage preparations have demonstrated efficient bacterial killing and, in some cases, reduced bacterial colonization in experimental models [68,72]. However, these studies generally involved pathogenic *E. coli* strains other than AIEC and cannot be directly extrapolated to CD.

More directly relevant evidence has been obtained from studies specifically targeting AIEC. Titecat et al. [73] evaluated seven lytic bacteriophages against a large collection of AIEC strains and commensal bacterial isolates representative of the healthy intestinal microbiota. The resulting phage cocktail showed broad lytic activity against AIEC strains while exhibiting limited activity against the tested commensal bacteria. In experimental models of intestinal inflammation, phage administration was associated with reductions in inflammatory and histological parameters. These findings provide important preclinical proof-of-concept for the selective targeting of AIEC; however, they do not establish clinical efficacy in patients with CD.

Similarly, Galtier et al. [74] investigated bacteriophages targeting the LF82 strain, one of the best-characterized AIEC isolates associated with CD. In experimental models, selected phages reduced the bacterial burden of LF82, supporting the feasibility of targeting AIEC with highly specific bacteriophages. More recently, Chevarin et al. [23] evaluated the ECO ACTIVE phage cocktail against AIEC isolates obtained from patients with CD in France and Hong Kong. The cocktail demonstrated lytic activity against AIEC strains from both geographic populations, supporting the potential applicability of this approach across genetically and geographically distinct bacterial isolates. Nevertheless, these findings remain preclinical and require validation in well-designed human studies.

Limited evidence from human studies has also explored the effects of orally administered phage preparations targeting *E. coli*. For example, Fèbvre et al. [70] conducted a randomized, double-blind, placebo-controlled crossover study evaluating an *E. coli*-targeting phage cocktail in healthy adults. The intervention reduced fecal *E. coli* levels without major disruption of the overall gut microbiota. However, this study was not conducted in patients with CD and did not specifically target AIEC. Consequently, it should be considered evidence supporting the feasibility and tolerability of oral phage administration rather than evidence of therapeutic efficacy in CD.

Overall, the current evidence supporting phage therapy in CD remains predominantly preclinical. Studies specifically targeting AIEC provide a strong biological rationale for further investigation, particularly because phages may offer selective antimicrobial activity while potentially preserving the broader commensal microbiota.

To our knowledge, no phage-based therapy has yet been established as a standard treatment for CD or incorporated into major clinical guidelines. Future clinical studies should include careful characterization of the bacterial target, preferably identifying AIEC-positive or otherwise microbiologically defined patient populations, and should evaluate not only changes in bacterial abundance but also clinically relevant outcomes such as clinical remission, endoscopic response, biomarker changes, safety, and long-term microbiome effects.

### 2.4. Fecal Microbiota Transplantation (FMT)

**Figure 6:**
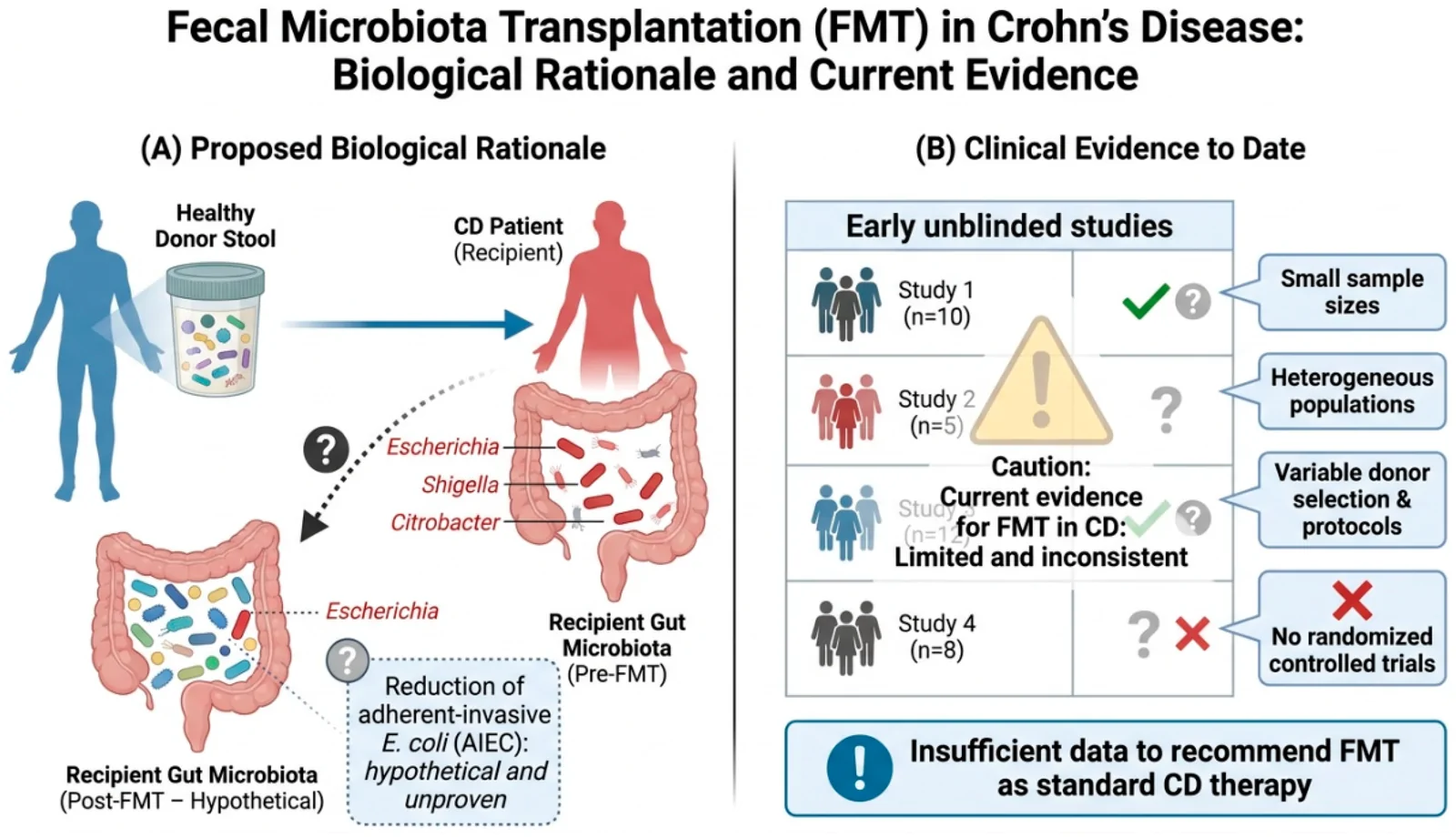
FMT: Restoring microbial diversity and correcting dysbiosis.

FMT has emerged as a potential microbiome-directed strategy for CD based on the evidence that dysbiosis and altered host–microbiota interactions may contribute to disease pathogenesis. FMT involves the administration of processed stool from a carefully screened healthy donor to a recipient, with the aim of modifying the intestinal microbial ecosystem (Figure 6).

Although FMT has demonstrated clinical efficacy in other gastrointestinal conditions, its therapeutic role in CD remains uncertain. Several studies have reported alterations in the intestinal microbiota of patients with CD, including reduced microbial diversity and enrichment of specific pathobionts. Species belonging to genera such as *Escherichia*, *Shigella*, and *Citrobacter* have been reported in association with CD in microbiome studies [75]. However, microbiome associations should not be interpreted as evidence that individual bacterial taxa represent direct therapeutic targets. In particular, the presence or relative abundance of *E. coli* does not necessarily indicate the presence of AIEC, which requires specific phenotypic or molecular characterization.

Early uncontrolled studies suggested that FMT may induce clinical improvement or remission in some patients with CD (Table 3) [76,77]. However, these studies were generally limited by small sample sizes, heterogeneous patient populations, variable donor selection procedures, differences in FMT preparation and administration, and the absence of appropriate control groups. Consequently, the findings of these early studies should be interpreted cautiously.

The systematic review by Fehily et al. [78] included 13 cohort studies and 2 randomized controlled trials, providing a mixed body of evidence on the efficacy of FMT for the induction of clinical and endoscopic remission in patients with active CD. Their analysis suggested that FMT was associated with higher rates of steroid-free remission compared with control interventions. Clinical response rates during early follow-up also appeared to be higher following multiple FMT administrations than after a single FMT administration. However, the substantial heterogeneity among the included studies should be taken into account when interpreting the reported findings.

The randomized pilot study by Sokol et al. [79] evaluated a single FMT administered by colonoscopy in patients with colonic or ileocolonic CD who had achieved clinical remission following corticosteroid treatment. Eight patients received FMT and nine received sham transplantation. The study did not achieve its predefined primary endpoint related to donor microbiota implantation. Nevertheless, steroid-free clinical remission rates were numerically higher in the FMT group at weeks 10 and 24, although the difference in flare-free survival did not reach statistical significance. A significant reduction in the Crohn’s Disease Endoscopic Index of Severity (CDEIS) was observed within the FMT group at week 6 but not within the sham group. These findings provide preliminary evidence of biological and potential clinical activity but are insufficient to establish the efficacy of FMT for maintaining remission in CD.

More recent randomized evidence has further highlighted the uncertainty surrounding the efficacy of FMT in CD. In a multicenter, double-blind, placebo-controlled trial in patients with mild-to-moderate CD, FMT was administered initially by colonoscopy followed by oral capsules. The study did not achieve its primary endpoint of combined clinical and endoscopic remission and was terminated early because of futility [80]. These findings further emphasize the need for caution when interpreting the positive results reported by earlier uncontrolled or small clinical studies.

Meta-analyses have reported variable estimates of clinical benefit. Cheng et al. [81] analyzed 12 studies and reported pooled clinical remission and clinical response rates of approximately 62% and 79%, respectively, following FMT. However, these pooled estimates should be interpreted cautiously because the included studies differed substantially in design, patient populations, disease activity, donor selection, FMT preparation, route of administration, treatment frequency, and definitions of clinical response and remission. Importantly, pooled response rates derived largely from uncontrolled studies do not provide the same level of evidence as placebo-controlled randomized trials.

Some studies have suggested that repeated or sequential FMT administration may prolong clinical response or delay relapses in selected patients. Li et al. [82] evaluated FMT in patients with CD who had previously experienced treatment failure or intolerance to infliximab and reported clinical improvement in a proportion of participants. Although these findings are of interest, the study design and limited sample size preclude definitive conclusions regarding the efficacy of FMT as a treatment strategy after anti-TNF failure. Accordingly, the term “switch strategy” should be avoided unless supported by comparative randomized evidence. Zhang et al. [83] reported a case of severe, refractory, fistulizing CD successfully treated with a single FMT. The patient achieved clinical remission one month after FMT, with the Crohn’s Disease Activity Index (CDAI) decreasing from 537 at baseline to 143 and maintained clinical remission during 9 months of follow-up. Although this single case provided preliminary support for the potential efficacy of FMT in refractory CD, its findings cannot be generalized to larger patient populations.

**Table 3:** Clinical studies evaluating FMT in patients with CD.

Study	Design	*n*	Disease/Severity	Pre-FMT Antibiotics	Clinical Outcome	Follow-Up
Cui et al. [76]	Cohort	30	CD/Moderate–severe	None	Remission: 23/30; Response: 26/30	6–15 months
Sokol et al. [79]	RCT	17	CD/Remission	None	Remission at week 10: 7/8 vs. 4/9	24 weeks
Gordon et al. [84]	Case report	1	CD/Severe	Vancomycin for concomitant CDI	Response: 1/1	6 months
Suskind et al. [85]	Cohort	9	CD/Mild–moderate	Rifaximin for 3 days	Remission: 5/9 at weeks 6 and 12	12 weeks
Vaughn et al. [86]	Cohort	19	CD/Active	None	Remission: 10/19; Response: 11/19	26 weeks
Goyal et al. [87]	Cohort	4	CD/Mild–moderate	Metronidazole/vancomycin for 5 days	Remission: 2/4; Response: 3/4	6 months
Bak et al. [88]	Case report	1	CD/Active	None	Remission: 1/1	12 months
Gutin et al. [89]	Cohort	10	CD/Active	Ciprofloxacin for 5 days	Remission: 1/10; Response: 3/10	12 months

Kong et al. [77] analysed metagenomic data from patients with CD who received FMT while in remission, assessing changes in the gut microbiome at both the species and strain levels before and after FMT. They identified FMT-induced changes in specific bacterial taxa that were associated with subsequent disease relapse. These findings provide mechanistic insights into the relationship between microbiota changes following FMT and clinical outcomes, but they do not establish a causal relationship between specific microbial changes and disease recurrence. The mechanisms through which FMT might influence CD remain incompletely understood. Potential effects include modification of microbial diversity, restoration of microbial metabolic functions, modulation of host immune responses, and changes in disease-associated microbial communities. However, direct evidence demonstrating that FMT induces sustained remission through the eradication of AIEC is currently lacking. Therefore, any potential reduction in AIEC or other pathobionts should be considered a hypothetical mechanism rather than an established explanation for the clinical effects of FMT. Safety is another important consideration. Although many studies have reported that FMT is generally well tolerated, the procedure carries potential risks related to transmission of infectious agents and other donor-derived factors. Rigorous donor screening and standardized processing protocols are therefore essential. Differences in donor selection, stool preparation and storage, route of administration, and treatment frequency also represent major sources of variability among published studies. Overall, FMT remains an investigational therapeutic strategy for CD. The available evidence includes promising signals from uncontrolled studies and small clinical trials, but randomized evidence remains limited and inconsistent. At present, the available data are insufficient to establish FMT as a standard treatment for the induction or maintenance of remission in CD. Future adequately powered, multicenter randomized controlled trials should use standardized protocols and incorporate clinically meaningful outcomes, including clinical and endoscopic remission, together with mechanistic endpoints related to microbiota engraftment and function. Identification of patient subgroups most likely to benefit from FMT, together with standardized donor selection and administration protocols, will be essential to define the future therapeutic role of FMT.

## 3. Nutraceuticals for CD: Therapeutic Potential and Current Role

Nutraceutical and dietary interventions have attracted increasing interest as potential complementary approaches in CD. These compounds may influence inflammatory pathways, oxidative stress, epithelial barrier function, and host–microbiota interactions. However, the available evidence is highly heterogeneous, and results obtained in experimental models or small clinical studies should not be interpreted as establishing therapeutic efficacy. At present, nutraceutical interventions should generally be considered complementary rather than alternatives to established medical therapies for CD.

### 3.1. Polyphenols and Curcumin

Polyphenols are plant-derived compounds with antioxidants and immunomodulatory properties. Experimental studies suggest that some polyphenols may influence inflammatory signaling pathways, including NF-κB and mitogen-activated protein kinase (MAPK) pathways [90,91,92]. Curcumin, a polyphenolic compound derived from *Curcuma longa*, has been extensively investigated because of its potential anti-inflammatory effects, including modulation of NF-κB signaling and cytokine production [93].

Clinical evidence for curcumin in CD remains limited. A randomized, double-blind, placebo-controlled study conducted in Japan evaluated Theracurmin^®^, a highly bioavailable curcumin formulation, in patients with mild-to-moderate CD. Treatment was associated with improvements in clinical disease activity and endoscopic parameters compared with placebo [94]. Although these findings are encouraging, the relatively limited number of available studies and the heterogeneity among curcumin formulations currently preclude firm conclusions regarding its routine use in CD. Larger randomized controlled trials are required to confirm efficacy, identify optimal formulations and doses, and determine which patients may benefit most.

### 3.2. Boswellia serrata

Extracts of *Boswellia serrata* have also been investigated because of their potential anti-inflammatory properties. Early clinical studies suggested that *Boswellia serrata* extracts may reduce disease activity in patients with active CD. In one clinical trial, treatment with a *Boswellia* extract was reported to produce improvements in disease activity comparable to those observed with mesalamine [95]. However, evidence supporting a role in maintaining long-term remission is less convincing. A subsequent longer-term study did not demonstrate a significant benefit of *Boswellia serrata* compared with placebo for the maintenance of remission [96]. Therefore, although *Boswellia serrata* may have potential as a complementary intervention, the current evidence is insufficient to support its routine therapeutic use in CD.

### 3.3. Artemisia absinthium

The herbal preparation *Artemisia absinthium* (wormwood) has been evaluated in small clinical studies, primarily because of its potential anti-inflammatory and steroid-sparing effects. In patients undergoing corticosteroid tapering, treatment with wormwood preparations was associated with improvements in clinical disease activity in some studies [97,98]. Reductions in circulating TNF-α concentrations have also been reported. However, the available studies involved relatively small patient populations, and differences in formulations and treatment protocols limit the generalizability of the findings. Consequently, *Artemisia absinthium* remains an investigational complementary approach requiring confirmation in larger, well-controlled trials.

### 3.4. Cannabis-Derived Compounds

Cannabis-derived compounds, including cannabidiol (CBD) and Δ9-tetrahydrocannabinol (THC), have been investigated for their potential effects on symptoms and quality of life in patients with CD. Some clinical studies have reported improvements in clinical symptoms and disease activity indices following treatment with cannabis-based preparations [99]. However, these improvements have not consistently been accompanied by significant changes in objective markers of inflammation or endoscopic disease activity. Therefore, the currently available evidence does not support cannabis-derived compounds as disease-modifying treatments for CD, although they may influence symptom perception in selected settings.

### 3.5. Dietary Fiber and Microbiota-Derived Metabolites

Dietary fiber represents another area of interest because it can serve as a substrate for microbial fermentation and the production of short-chain fatty acids (SCFAs), including butyrate. SCFAs contribute to epithelial barrier function and influence intestinal immune responses [100,101]. However, the effects of fiber-based interventions may depend on disease location, inflammatory activity, intestinal strictures, baseline dietary habits, and the composition and functional capacity of the intestinal microbiota. Therefore, the biological rationale supporting fiber consumption should not be interpreted as evidence for a uniform therapeutic effect in all patients with CD.

### 3.6. Vitamin D and Zinc

Micronutrient deficiencies are common in patients with CD and may be associated with disease severity, impaired nutritional status, and adverse clinical outcomes. Vitamin D deficiency has been associated with increased disease activity and unfavorable outcomes in observational studies [102]. In a small open-label study, vitamin D_3_ supplementation was associated with improvements in CDAI and quality-of-life scores after 24 weeks [103]. However, these findings do not establish vitamin D supplementation as a primary anti-inflammatory treatment for CD. Its most clearly established role remains the identification and correction of deficiency, while the potential benefits of supplementation beyond correction of deficiency require further investigation.

In a small study involving patients with quiescent CD, zinc supplementation improved alterations in intestinal permeability [104]. Zinc deficiency has been associated with impaired epithelial barrier function and unfavorable clinical outcomes in IBD [105]. Observational evidence suggests that normalization of zinc deficiency may be associated with improved clinical outcomes [105]. Nevertheless, these findings should be interpreted cautiously because of the limited size and design of the available studies. Zinc supplementation should therefore primarily be considered in the context of documented deficiency or increased nutritional requirements rather than as an established anti-inflammatory treatment.

### 3.7. Probiotics

The role of probiotics in CD remains controversial. Unlike ulcerative colitis and pouchitis, probiotics have not consistently demonstrated efficacy for the induction or maintenance of remission in CD. Early studies of *Saccharomyces boulardii* and *Lactobacillus rhamnosus* GG reported potentially beneficial effects in selected patient populations [106,107]. However, these findings were not consistently confirmed in subsequent or larger clinical trials [108,109]. Therefore, the current evidence does not support the routine use of probiotics for induction or maintenance of remission in CD.

### 3.8. Palmitoylethanolamide and Omega-3 Fatty Acids

Palmitoylethanolamide (PEA) is an endogenous lipid mediator that has been investigated for its potential effects on intestinal inflammation, epithelial barrier function, and abdominal pain. Experimental and clinical studies in gastrointestinal disorders suggest that PEA may modulate peroxisome proliferator-activated receptor-α (PPAR-α) signaling and influence enteric glial and immune responses [110,111]. However, evidence supporting its therapeutic efficacy specifically in CD remains limited, and further clinical studies in IBD are required. Similarly, n-3 polyunsaturated fatty acids (PUFAs) have been investigated because of their potential effects on inflammatory mediators. Although experimental and biological data provide a rationale for their use, randomized clinical trials evaluating omega-3 supplementation for the maintenance of remission in CD have not demonstrated a consistent preventive effect on relapse [112,113]. Therefore, n-3 PUFA supplementation cannot currently be considered an established strategy for maintaining remission in CD. Overall, nutraceutical and dietary interventions represent promising complementary approaches for addressing inflammation, oxidative stress, epithelial barrier dysfunction, and nutritional deficiencies in CD. However, the strength of evidence differs substantially among individual interventions. Some compounds, including curcumin and selected herbal preparations, have shown encouraging results in small clinical studies but require confirmation in larger randomized controlled trials. In contrast, vitamin D and zinc supplementation have an established role in the correction of documented deficiencies, although their ability to directly modify the course of CD remains uncertain. Probiotics, PEA, n-3 PUFAs, and cannabis-derived compounds currently have limited or inconsistent evidence as disease-modifying therapies. Therefore, nutraceutical interventions should not replace established medical treatments and should be evaluated according to the individual patient’s nutritional status, disease characteristics, concomitant therapy, and the quality of the available evidence.

## 4. Conclusions

Collectively, emerging microbiome-directed strategies—including selected antibiotic approaches, AMPs, gut bacteriophages, FMT, and nutraceutical interventions—represent promising complementary avenues for the management of patients with active CD. However, these approaches differ substantially in their mechanisms of action, stage of development, and level of supporting evidence. A common rationale underlying these interventions is the modulation of host–microbe interactions, including the targeting of disease-associated pathobionts such as AIEC, restoration of microbial homeostasis, and modulation of epithelial barrier and immune function. Nevertheless, the association of AIEC and other microbial alterations with CD should not be interpreted as definitive evidence of a causal role, and the relevance of microbiome-directed interventions may vary according to individual patient characteristics.

Overall, these emerging therapeutic modalities highlight the potential value of a precision medicine framework integrating microbial, immunological, and clinical characteristics. Advances in biomarker discovery and molecular profiling may contribute to improved patient stratification and the identification of individuals most likely to benefit from specific therapeutic approaches. However, the clinical utility of predictive biomarkers for guiding microbiome-directed interventions remains to be established.

Future research should prioritize adequately powered, multicentre randomized controlled trials using standardized methodologies and clinically meaningful endpoints. The integration of microbial characterization with host immune profiling and, where appropriate, multi-omics approaches—including metagenomics, proteomics, and metabolomics—may help identify biologically and clinically defined patient subgroups. Such studies will be essential to determine therapeutic efficacy, optimize treatment protocols, and clarify the role of microbiome-directed strategies in different clinical settings. As our understanding of host–microbiome interactions continue to evolve, some of these approaches may eventually become clinically applicable adjuncts to established therapies. At present, however, they should primarily be considered investigational or complementary strategies rather than alternatives to biologic or advanced small-molecule treatments. 

## Figures and Tables

**Table 1:** Nitroimidazoles, ciprofloxacin and rifaximin for prophylaxis of post-operative CD recurrence.

Study	Study Design and Population	Intervention	Clinical Recurrence	Endoscopic Recurrence	Safety/Main Conclusions
Rutgeerts [24]	Double-blind RCT; 60 patients after ileal resection	Metronidazole 20 mg/kg/day for 3 months vs. placebo	4% vs. 25% at 1 year (*p* = 0.04)	13% vs. 43% at 3 months (*p* = 0.02)	Reduced early endoscopic and clinical recurrence. Adverse effects limited treatment tolerability.
Rutgeerts [25]	Double-blind RCT; 80 patients after curative ileocolonic resection	Ornidazole 1 g/day vs. placebo	7.9% vs. 37.5% at 12 months; OR 0.14 (95% CI 0.037–0.546), *p* = 0.0046	53.6% vs. 79% at 12 months; OR 0.31 (95% CI 0.10–0.94), *p* = 0.037	Significant reduction in both clinical and endoscopic recurrence. Adverse events significantly increased treatment withdrawal.
D’Haens [26]	RCT; 81 high-risk patients after ileocecal resection	Metronidazole for 3 months + azathioprine vs. metronidazole + placebo	No significant difference	55% vs. 78% at 12 months, *p* = 0.035	Supports the use of short-term metronidazole as part of combination postoperative prophylaxis.
Doherty [27]	Cochrane systematic review/meta-analysis	Metronidazole or ornidazole vs. placebo/no treatment	RR 0.23 (95% CI 0.09–0.57); NNT ≈ 4	RR 0.44 (95% CI 0.26–0.74); NNT ≈ 4	Consistent benefit for both outcomes, but adverse events increased (RR 2.39, 95% CI 1.54–3.70).
Chen [28]	Systematic review and network meta-analysis; 45 RCTs	Nitroimidazoles vs. placebo/other strategies	RR 0.35 (95% CI 0.14–0.84); GRADE: High; SUCRA 0.77	No significant benefit demonstrated	Confirms benefit for clinical recurrence, but suggests lower efficacy than several biologic strategies.
Shehab [29]	Systematic review and network meta-analysis; 42 studies	Multiple postoperative strategies, including metronidazole	Antibiotics less effective than anti-TNF therapies	Anti-TNF therapies ranked among the most effective strategies	Supports a more selective role for antibiotics in the current postoperative treatment landscape.

**Table 2:** Nitroimidazoles, ciprofloxacin and rifaximin for active CD: evidence from randomized trials and meta-analyses.

Study	Antibiotics	Duration	Clinical Remission	Overall Evidence
Steinhart et al. [30]	Metronidazole + ciprofloxacin ± budesonide	8 weeks	OR 1.02	No significant benefit
Rahimi et al. [31]	Metronidazole, ciprofloxacin, cotrimoxazole ± combinations	2–24 weeks	OR 2.26	Benefit vs. placebo
Prantera et al. [32]	Rifaximin	12 weeks	NR	Efficacy not clearly established
Wang et al. [33]	Ciprofloxacin, metronidazole, rifaximin, clarithromycin	2–16 weeks	OR 1.35	Modest benefit
Su et al. [34]	Fluoroquinolones, clarithromycin, metronidazole, rifaximin	≥4 weeks	OR 1.35	Moderate benefit
Townsend et al. [35]	Rifaximin, clarithromycin, metronidazole, cotrimoxazole, anti-MAP ± budesonide	6–14 weeks	OR 0.33–0.77	No consistent benefit

NR, not reported; OR, odds ratio; CDAI, Crohn’s Disease Activity Index.

## Data Availability

No new data were created or analyzed in this study. Data sharing is not applicable to this article.

## Abbreviations

The following abbreviations are used in this manuscript:

AIEC	Adherent-invasive *Escherichia coli*
AMPs	Antimicrobial peptides
CD	Crohn’s disease
IBD	Inflammatory bowel disease
IECs	Intestinal epithelial cells
MAP	*Mycobacterium avium* subspecies *paratuberculosis*
FMT	Fecal microbiota transplantation
SCFAs	Short-chain fatty acids
TNF-α	Tumor necrosis factor-alpha
IFN-γ	Interferon-gamma
IL	Interleukin
NF-κB	Nuclear factor kappa-light-chain-enhancer of activated B cells
MAPK	Mitogen-activated protein kinase
CEACAM6	Carcinoembryonic antigen-related cell adhesion molecule 6
VAT-AIEC	Vacuolating autotransporter toxin of adherent-invasive *Escherichia coli*
DSS	Dextran sulfate sodium
CDAI	Crohn’s Disease Activity Index
SES-CD	Simple Endoscopic Score for Crohn’s Disease
CDEIS	Crohn’s Disease Endoscopic Index of Severity
RCTs	Randomized controlled trials
AMP	Antimicrobial peptide
HNP-1	Human neutrophil peptide-1
PPARα	Peroxisome proliferator-activated receptor alpha
PUFAs	Polyunsaturated fatty acids
CBD	Cannabidiol
THC	Δ9-Tetrahydrocannabinol
AI	Artificial intelligence
SHAP	SHapley Additive exPlanations
DNA	Deoxyribonucleic acid
RNA	Ribonucleic acid
EU	European Union
USA	United States of America
